# Assessment of hospitalized patients awareness of their rights: a cross-sectional descriptive study

**DOI:** 10.11604/pamj.2021.38.157.24824

**Published:** 2021-02-11

**Authors:** Dildar Muhammad, Anayat Jan, Sehrish Naz

**Affiliations:** 1 Institute of Nursing Sciences, Khyber Medical University, Peshawar, Pakistan

**Keywords:** Assessment, awareness, hospital, hospitalized patients, patients’ bill of rights

## Abstract

**Introduction:**

in this modern era and in the speedily changing atmosphere of health care, health care practice and patients has affected by many factors. These days, in many states the patient rights has become the pivot of the national attention in medical culture. Awareness of health rights is important to achieve the best level of health care. The study was aimed to assess the awareness of hospitalized patients about the rights.

**Methods:**

a cross-sectional descriptive study was conducted in different wards of public sector tertiary care hospitals of Peshawar, over four months duration from February to May 2018. Hospitalized patients who were being admitted for at least two days from 17 to 70 years of age were included. Before asking the patients to answer the questionnaire, consent from the patients was acquired. A self-administered semi-structured questionnaire was adopted. Data analysis was done through SPSS version 22.

**Results:**

among 200 participants 46% were male and 54% were female, 35.5% were from urban and 64.5% were from rural areas. Patients were less aware of their individual rights, like 65.5% of clients were not cognizant of the patients special bill of rights while 59% were aware to receive non-discriminatory and timely health services.

**Conclusion:**

most of the clients were not conscious of their individual health rights. About half of the sample knew that the patients will receive respectful care and the patients will receive care in clean and medically safe environment.

## Introduction

By birth human beings are free and have similar rights and are equally graceful. None of the human being shall be entitled to brutality, immoral and humiliating treatment and penalty. Furthermore, none of the individual would be subjected to any medical or scientific examination without his sweet will [1]. In this advance age of technology, respect for the patients values and preferences, issues about the patients´ choices and availability of nursing care are becoming more complex. With rising cost of health care the medico-legal issues and the patients´ awareness are increased, health professionals are now more accountable to the public than ever before [2]. Patients want to be delivered with best treatment and to take part in taking judgment, planned procedure or treatment and their manifold reciprocal. As a whole, consciousness about the human rights has been on the rise [3].

A physician should always act in the best interest of the patients. Similar steps must be taken to make sure, patients autonomy and justice. Physician and other persons or individuals taking part in health process have mutual responsibility to acknowledge and maintained these rights [4]. Awareness among people generally and in patients particularly is not parallel with advancement of information technology. A study conducted by Kerman University of Medical Sciences (Iran) in 2013 show that 30.5% of patients had low level of consciousness, 59.4% of them had moderate and 10.1% had good level of consciousness about their rights [5]. A study carried out in Dhaka (Bangladesh) on patients´ awareness of their rights reveals 59% of all the participants had good level of awareness about their rights [6].

Patients´ rights execution do not bound the skillful management of medicine. On contrast it influences the modernization of health care practices and an equal distribution of obligation can be acquired between patient, physician and nurse, if the special bill of patients´ rights is implemented. In a cross-sectional study conducted in Sudan a high number of participants 93%, were not informed of their rights of patients [7]. Another study carried out in Iran show that 45.2% of patients had a good awareness related to patients´ rights charter and 6.4% were poor awareness [8].

The Pakistan medical and dental council (PMDC) code of ethics has declared the patient rights as right to receive information from physician, obtain copies or summaries of their medical records, make decision, respect and dignity etc. [9]. A study conducted in Punjab, Pakistan, demonstrate that only 36% of all the participants were conscious about their rights [10]. With reference to Pakistan, it was a highly informative study because a very little work has done on this topic, so this study will fill the gap in literature and will draw the attention of the society towards this issue and will provide a background for further research on this topic. This study was carried out to assess the awareness of hospitalized patients about their rights.

## Methods

This was a descriptive cross sectional survey conducted through close ended questions and was carried out in two tertiary care governmental hospitals in Peshawar, Pakistan, Khyber Teaching Hospital (KTH) and Hayatabad medical complex (HMC), Peshawar, over four months duration from February to May 2018, investigating awareness of rights among hospitalized patients. The study sample consisted of 200 hospitalized patients. Through systematic random sampling, 100 patients were selected from KTH and 100 were selected from HMC. The questionnaire was taken from the similar study conducted in Punjab, Pakistan and was self-developed structured questionnaire [10]. The rights declared by PMDC in code of ethics were also included. The self-developed questionnaire was validated by two experts of the field.

The questionnaire was translated into national language (Urdu) for the sake of easily understand by the participants while it was structured, developed and finalized in English. The questionnaire was designed in two parts: socio-demographic information such as, age, sex, marital status etc. was included in the first part; while the questions regarding awareness of patient´s rights such as receiving non-discriminatory and timely care, to be kept fully informed of diagnosis and treatment plan, right of being respected etc. designed the second part of the questionnaire. Questions regarding awareness of patient about their rights required a yes, no and to some extent options.

Data was collected by the author´s (students of bachelor of nursing sciences 2018). The participants were approached by the author in person. They were introduced with the study through a brief description and explaining the aim of the study to them and before asking the participants to answer the questions their consent was taken by the researcher. The participants were assured that their privacy, anonymity of their information and their identities would be kept confidential. The response rate of the patients was 100% and no one refused to participate. The study was conducted on all conscious patients and all the patients were capable of giving their consent. In this study only those patients were included who had stayed in hospital for at least 2 days while unstable patients, pediatrics patients, patients from the highly intensive care unit, cardio-vascular care unit and patients from the high dependency ward were not included.

The study was approved by the faculty of Institute of Nursing (Khyber Medical University) and by the administration of Hayatabad Medical Complex and the Khyber Teaching Hospital Peshawar. The statistical package for social sciences version 22 (SPSS) was used for the statistical analysis of this study.

## Results

A total of 200 patients were questioned. Table 1 shows the demographic profile of the study population. Of the total sample 92 (46%) were males and 92 (46%) were females. The age range was from 17 to 70 years. With regard to education, 38% were uneducated, 12% had primary education, 8% had middle school education, 20% had matric, 12% had intermediate and 10% had graduated and above graduation (Table 1). Individual questions were asked with regard to different rights of the patients, 65.5% were not conscious about the patients special bill of rights in the hospital, 59% were not aware that the patients will receive non-discriminatory and timely health services. About 54.5% were not aware that the patients would be provided access to nurse/doctor and 53.5% were not aware that the patient´s beliefs would be respected and 54% were not aware that the diagnosis and treatment modality would be explained to the patients. Sixty two percent of the participants were not aware that the treatment options will be discuss with the patients, 53.3% were not aware to consult with another doctor for second opinion (Table 2).

**Table 1:** socio-demographic characteristics of all the participated patients in the study

Characteristics	Frequency	Percentage (%)
**Gender**		
Male	92	46
Female	108	54
**Age**		
Less than 20	32	16
From 20 to 39	92	46
From 40 to 59	50	25
Over 60	26	13
**Education level**		
Uneducated	76	38
Primary	24	12
Middle	16	8
Matric	40	20
Intermediate	24	12
Graduation and above	20	10
**Place of residency**		
Urban	71	35.5
Rural	129	64.5
**Income level**		
Less than 20000	32	16
21000 to 40000	56	28
41000 to 60000	46	23
61000 to 100000	33	16.5
110000 and above	24	12
Not mentioned	9	4.5
**Occupation**		
Business-man	23	11.5
House-wife	72	36
Service-holder	31	15.5
Student and other	74	37
**Marital status**		
Married	132	66
Un-married	58	29
Widow	8	4
Divorce	2	1
**Length of stay in hospital**		
Less than one week	80	40
One week to 2 weeks	83	41.5
Two weeks to 1 month	26	13
More than 1 month	11	5.5
**Number of hospitalization admission**		
None	20	10
Once	50	25
Twice	57	27.5
Three times	42	21
More than 3 times	31	15.5

**Table 2:** patients' rights and their assessment of awareness about their rights

No	Rights of the hospitalized patients comprised the following rights	Aware frequency (%)	To some extent frequency (%)	Not aware frequency (%)
1	Patient's special bill of rights in the hospital	49 (24.5)	20 (10)	131 (65.5)
2	The patient will receive non-discriminatory and timely health services	57 (28.5)	25 (12.5)	118 (59)
3	To receive respectful care	89 (44.5)	20 (10)	91 (45.5)
4	The specialist will examine the patient and the competent staff will provide care	79 (39.5)	23 (11.5)	98 (49)
5	The patients will be kept fully aware of their diagnosis and treatment plan	63 (31.5)	28 (14)	109 (54.5)
6	To take informed consent for any medical interventions the patients will receive all essential information	60 (30)	28 (14)	112 (56)
7	In case of refuse treatment, the effects would be explained to the patients	78 (39)	36 (18)	86 (43)
8	Patients would be instructed about their treatment in understandable language	73 (36.5)	26 (13)	101 (50.1)
9	Confidentiality and privacy regarding the patient's information's would be assured to them	61 (31.5)	32 (16)	107 (53.5)
10	Appropriate medications, follow up appointment and required information would be explained to the patients as per doctor recommendation to be discharged	73 (31.5)	21 (10.5)	106 (53)
11	Medical report summarizing patients' medical condition and course during admission would be handed over to the patients	47 (23.5)	28 (14)	125 (62.5)
12	To be informed of duration of treatment	59 (29.5)	34 (17)	107 (53.5)
13	To provide access to nurse/doctor	62 (31)	29 (14.5)	109 (54.5)
14	To respect the patients' beliefs	62 (31)	31 (15.5)	107 (53.5)
15	Clean and medically safe environment will be provided to receive best care	97 (48.5)	27 (13.5)	76 (38)
16	The diagnosis and treatment modality would be explained to the patient	63 (31.5)	29 (14.5)	108 (54)
17	The treatment option will be discus with patient	51 (25.5)	25 (12.5)	124 (62)
18	Satisfaction with the current practices of patient's rights in the hospital	49 (24.5)	55 (27.5)	96 (48)
19	Clients can consult with another doctor for second opinion	73 (36.5)	30 (15)	97 (53.5)

## Discussion

Patients´ rights implementation contributes to the advancement of health care practices and in making patients, physician and nurse conscious about their individual responsibility [3]. It is mandatory for a country to understand various existing health care system and make them well organized for comprehending such phenomena the patient´s awareness about their rights is necessary [11]. Up to some extent human basic rights are also comprised of patients´ rights. In the field of medical sciences and in the nursing practices, knowledge about the patient´s rights has not diverted the attention of the health care professionals and of ordinary people in the medical culture in the modern world [12].

This study indicates that the awareness of patients about their right was low. In our study, 65.5% of the total participants were not vigilant about the special bill of patients´ rights in the hospital however this level of awareness is much higher as compare to a study conducting in Sudan which show that 95.4% of patients were not aware that the Sudanese federal ministry of health (FMoH) had published a patients´ bill of rights [10]. In a similar study conducted in Saudi Arabia, 74.8% of participants were not aware of the patients´ bill of rights [3]. In addition, in a study carried out in Iran, 58.05% of the patient´s awareness about the special bill of patients´ rights was low [13]. Another study conducting in Iran indicates that 45.2% of patients had good awareness related to patient´s rights charter and 6.4% were poor [8]. Number of factors may be found to be contributing to this low level of awareness such as: in this study we have a very large number of patients uneducated (38%), which may be a major contributing factor in the unawareness of patients about their rights.

In this study, 53.5% of patients were not aware of privacy and confidentiality right. Moreover, 31% of patients were aware that their beliefs would be respected. A study conducted in Egypt show that 89.3% of the child guardian were aware that the physician should respect the privacy of the patients [14]. Physician-patient interaction should remain confidential. The physician should not disclose the patient secret information´s and records unless at the request of the patient or required by law [15]. American medical association privileged the patient to decide their treatment or any procedure by themselves [11]. In this study only, 30% of patients were aware that the patients will know about all the obligatory information to express their will for consent, but in a study conducted in Rawalpindi, Pakistan, show 46.3% awareness among patients [11].

This study show that 48% of the total participants were not complacent from the current practices of patients´ rights in the hospital. On the other hand in Iran, the situation is much good as 57.5% of patients were complacent from the performance of the health care personnel with their respective rights [16].

Future directions: according to the entire study findings we would recommend that further qualitative exploratory studies should be carried out to know patients´ views and opinions because in our study most of the educated patients were known to their rights but were unable to get them during hospital study. Many patients wanted to tell their views but our study questionnaire was structured which was a major limitation of the study so that we would like to address this concern in the form of exploration of the phenomena. This will help the researchers to find up to what extent educated as well as non educated people know about their rights and if they don´t know or don´t able to get their proper rights then it is a further room for the research to find out the contributing factors behind their concerns. Furthermore, healthcare professionals should also be interviewed for their opinions and suggestions that if they have had knowledge about bill of patients´ rights then what are factors behind no implementation into the clinical practice.

## Conclusion

Majority of the participants were not vigilant about their rights while less than half were aware that the they have the right to receive care in a clean and medically safe environment. In addition, many of the patients were uneducated which may be a major contributing factor in the unawareness of their rights. The state has the responsibility to provide quality education, strictly implement the health policies and distribute special bill of patients´ rights among the masses. Demands, suggestions and recommendations of the patients should be listened and should be further preceded in the patient´s affairs department.

### What is known about this topic

- The Pakistan medical and dental council (PMDC) code of ethics has declared the patient rights as: right to receive information from physician; obtain copies or summaries of their medical records; make decision and respect and dignity.

### What this study adds

- Study adds that more than half of the patients were unknown to their rights;
- If majority is uneducated then audio or video teaching plans should be designed for them to make them aware about their rights;
- Proper policies should be designed to keep check on health professionals to entertain patients according to ethical rules and principles.
